# The symptom experience of people living with HIV and AIDS in the Eastern Cape, South Africa

**DOI:** 10.1186/1472-6963-8-271

**Published:** 2008-12-22

**Authors:** Karl Peltzer, Nancy Phaswana-Mafuya

**Affiliations:** 1 Health Systems Research Unit, Social Aspect of HIV/AIDS and Health, Human Sciences Research Council, Pretoria, Port Elizabeth, South Africa; 2 Department of Psychology, University of the Free State, Bloemfontein, South Africa; 3 Faculty of Community Health Sciences, University of the Western Cape, Bellville, South Africa

## Abstract

**Background:**

Symptom management for persons living with HIV (PLHIV) or AIDS is an important part of care management. Limited information about symptom prevalence exists about HIV infected persons in South Africa, in particular in the context of antiretroviral treatment (ART). The aim of this study was to assess HIV symptoms and demographic, social and disease variables of people living with HIV in South Africa.

**Methods:**

In 2007 607 PLHIV, sampled by all districts in the Eastern Cape Province and recruited through convenience sampling, were interviewed by PLHIV at health facilities, key informants in the community and support groups.

**Results:**

Two-thirds of the PLHIV (66%) classified themselves with being given an AIDS (advanced stage of HIV) diagnosis, 48% were currently on ART, 35% were currently on a disability grant for HIV/AIDS and for 13% the disability grant had been stopped. Participants reported that on the day of the interview, they were experiencing an average of 26.1 symptoms out of a possible 64. In a regression model with demographic and social variables, higher HIV symptom levels were associated with lower educational levels, higher age, urban residence and not on a disability grant, lack of enough food and having a health insurance, and in a regression model with demographic, social and disease variables only being on ART, lack of enough food and having a health insurance were associated with HIV symptoms.

**Conclusion:**

Symptom assessment provides information that may be valuable in evaluating AIDS treatment regimens and defining strategies to improve quality of life. Because of the high levels of symptoms reported, the results imply an urgent need for effective health care, home- and community-based as well as self-care symptom management to help patients and their families manage and control AIDS symptoms.

## Background

HIV and AIDS is one of the main challenges facing South Africa today. Of approximately 39.5 million people living with HIV worldwide in 2006, more than 63% were estimated to be from sub-Saharan Africa [1]. About 5.54 million people were estimated to be living with HIV in South Africa in 2005, with 18.8% of the adult population (aged 15–49) and about 12% of the general population infected. Women are disproportionately affected, accounting for approximately 55% of HIV-positive people in South Africa [2]. Women in the age group 25–29 are the worst affected, with HIV prevalence among them as high as 40%, while among men peak prevalence occurs in older age groups [2].

Although prevention still remains a top public health priority, the country has also recently (2003) instituted antiretroviral therapies (ART) in the public health sector. While AIDS still remains as the top cause of mortality for the country, the availability of ART means that HIV as a disease will likely be transformed from a high mortality acute infectious disease to a chronic disease. Persons with HIV and AIDS will continue to live longer, and will continue to pursue normal activities of daily living such as eating, drinking, recreation, and procreation. It is estimated that in the Eastern Cape Province of South Africa 21 294 Persons Living with HIV adults and 1 220 paediatrics were on ARV therapy in 2006 [3].

Wilson and Cleary [4] described a symptom as a person's perception of an abnormal physical, emotional, or cognitive state. Symptoms are perceived indicators of change in normal functioning as experienced by the person [5]. Persons infected with HIV report myriad symptoms that indicate disease progression. For example, in a study of 134 persons with HIV/AIDS in Taiwan, Tsai, Hsiung and Holzemer [6] reported that the following symptoms were frequently described: nausea (40.3%), pain (24.6%), anxiety (21.6%), vomiting (16.4%), diarrhoea (14.9%), fear (14.2%), and lack of appetite (10.4%). Fantoni et al. [7] found that the most frequent symptoms of the 1,128 HIV-infected persons they surveyed in Italy were fatigue (65%), anorexia (34%), cough (32%), pain (29%), and fever (29%). Mathews et al. [8] reported on the symptoms of a national sample of more than 4,000 HIV/AIDS outpatients in the United States: fever/night sweats (51.3%), diarrhoea (51%), nausea/anorexia (49.8%), dysesthesias (48.9%), and severe headache (39.3%). Karus et al. [9] found in their study of ill HIV patients (≥ 95% AIDS) in Alabama (*n *= 47), Baltimore (*n *= 91), and New York City (*n *= 117) that on average, patients reported 10.9 (SD = 7.6) to 12.7 (SD = 6.2) symptoms.

Makoae et al. [10] studied the symptom experience of 743 people living with HIV/AIDS in four countries in Southern Africa (Botswana, Lesotho, South Africa, Swaziland) in 2002 using the 64-item Revised Sign and Symptom Checklist for Persons with HIV Disease. Results indicate mostly unavailable ARV treatment, high levels of HIV symptoms and a strong correlation between the frequency of reported symptoms and their intensity. Participants who reported having enough money for daily expenses also reported significantly fewer symptoms. There were no significant differences in symptom frequency between men and women or by location of residence.

There is a paucity of more recent symptom prevalence data about HIV infected persons in South Africa, in particular in the context of ART. The aim of this study was to assess HIV symptoms and demographic, social and disease variables of people living with HIV in South Africa. Findings may ultimately aim to benefit PLHIV in South Africa.

## Methods

### Sample and procedure

The sample included 607 PLHIV sampled from all districts in the Eastern Cape Province: Amathole (81, 13.3%), Cacadu (83, 13.7%), Chris Hani (120, 19.8%), Nelson Mandela (80, 13.2%), OR Tambo (120, 19.8%), Alfred Nzo (82, 13.5%) and Ukhalamba (41, 6.8%). They were recruited through convenience sampling by other PLHIV at health facilities (42.0%), key informants in the community (44.1%) and support groups (13.9%). Fifteen locally based PLHIV (males and females) with a matric qualification were appointed and trained as data collectors to conduct face-to-face interviews in the districts. The 15 interviewers were asked to recruit respondents from their own local environment. Those interviewers who worked in a clinic or who were a member of a support group at a clinic consecutively approached PLHIV known to them at the different clinic sites. In case of recruitment through key informants, the interviewers contacted someone in their community who was well known to them. Also, the interviewers could recruit from their own support group in the community. The criteria for inclusion were PLHIV who were at least 18 years old and who were able to respond to an interviewer-administered questionnaire [11]. Informed consent was obtained from participants. The study was approved by the Human Sciences Research Council ethics committee and the Eastern Cape health department and conducted in 2007.

### Assessment measures

#### a) Demographic, social and health status characteristics

Information was gathered regarding participants' sex, age, race, years of education completed, residential area, medical aid status, degree of having had food, disability grant status, what month and year they tested HIV seropositive, whether they had been diagnosed with an AIDS case-defining condition, if they are currently taking antiretroviral medications, use of traditional medicine for HIV, self-reported CD4 cell counts, and frequency of hospitalization in the past three months.

#### b) The Revised Sign and Symptom Checklist for Persons with HIV Disease (SSC-HIVrev)

The SSC-HIVrev is a 72-item checklist of HIV/AIDS-specific physical and psychological symptoms and includes eight gynaecological problems not relevant to men [12]. Therefore the analysis was done on the 64 physical and psychological symptoms. The symptom-related survey items are scored using the following scale: 0 = not checked (not present today), 1 = mild, 2 = moderate, 3 = severe. Calculations included the total number of symptoms checked as present today (with a range of 0–64) and the total symptom intensity is a weighting of symptoms checked by the 1-to-3 rating of *mild, moderate, or severe *[12]. Validity and reliability of the instrument have previously been reported for a U.S. sample [12] and various African countries including South Africa [10]. Reliability estimates were calculated using Cronbach's alpha, which was .94 in this sample for the 64-item checklist.

### Data analysis

The data were analyzed using Statistical Package for the Social Sciences for Windows (Version 12.0) software (SPSS Inc, Chicago, IL). Basic statistical analyses were undertaken, such as descriptive statistics and cross-tabulations. Multiple hierarchical regression analysis with HIV symptom intensity as the dependent variable enabled comparison of demographic, social and disease variables with symptom frequency.

## Results

The participants (n = 607) reported that on the day of the interview, they were experiencing an average of 26.1 symptoms (SD = 13.7) out of a possible 64, and among women the total number of gynecological symptoms experienced today was 2.6 (SD = 2.4) out of a possible 8.

The frequency and intensity of the reported symptoms are presented in Table 1; headaches, fever, thirsty, fatigue, weakness, painful joints, nausea, muscle aches, fears and worries and dizziness were the top ten. Numbness/tingling of hands/fingers, weight gain in stomach area, loose stools and skinny arms and legs symptoms were significantly more frequently reported by PLHIV on ART than those not on ART, while diarrhoea, sore throat, painful swallowing, sore/bleeding gums and sore/bleeding gums were more frequently reported by those not on ART than those on ART. Typical possible side effects from ART such as rash (χ^2 ^= .09, *P *> .05), nausea (χ^2 ^= .11, *P *> .05) and vomiting (χ^2 ^= 1.21, *P *> .05) [13] did not differ significantly between patients on ART and not on ART.

**Table 1:** Symptom rank order, frequency, percentage and mean intensity

Problem	Rank	Frequency	Percentage	Mean intensity (a)
Headaches	1.	403	79.0	2.15
Fever	2.	351	68.8	1.83
Thirsty	3.	346	67.8	2.17
Fatigue	4.	340	66.7	2.10
Weakness	5.	338	66.3	1.79
Painful joints	6.	332	65.1	2.08
Nausea	7.	307	60.2	1.74
Muscle aches	8.	302	59.2	1.95
Fear/Worries	9.	300	58.8	1.87
Dizziness	10.	293	57.5	1.89
Lack of appetite	11.	281	55.1	1.80
Abdominal pain	12.	280	54.9	2.08
Depression (sadness)	13.	279	54.7	2.00
Numbness/tingling of legs	14.	272	53.3	1.93
Coughing/problems catching breath	15.	271	53.1	1.92
Concern over weight loss	16.	270	52.9	2.38
Night sweats	17.	269	52.7	2.03
Memory loss	18.	264	51.8	2.04
Constipation	19.	261	51.2	1.99
Chills (feeling very cold)	20.	258	50.6	1.89
Rash	21.	257	50.4	1.99
Itchy skin	22.	256	50.2	1.98
Anxious	23.	252	49.4	1.80
Numbness/tingling of arms	24.	249	48.8	1.86
Numbness/tingling of feet/toes	25.	244	47.8	1.92
Numbness/tingling of hands/fingers	26.	242(b)	47.5	1.83
Swollen feet	27.	241	47.3	2.03
Dry mouth	28.	240	47.1	2.02
Shortness of breath with activity	29.	240	47.1	1.84
Blurred vision	30.	238	46.7	2.18
Burning with urination	31.	237	46.5	2.04
Difficulty concentrating	32.	235	46.1	1.91
Day sweats	33.	232	45.5	1.87
Chest pain	34.	213	41.8	1.96
Heart racing	35.	210	41.2	2.11
Wheezing	36.	209	41.0	1.84
Insomnia/can't sleep	37.	201	39.4	1.84
Weight gain in stomach area	38.	195(b)	38.2	2.22
Diarrhoea	39.	193(c)	37.8	1.83
Easy bruising	40.	188	36.9	1.82
Shortness of breath at rest	41.	173	33.9	1.87
Loose stools	42.	167(b)	32.7	1.78
Gas/bloating	43.	162	31.8	1.72
Sore throat	44.	156(c)	30.6	1.80
White spots in mouth/Thrush	45.	156	30.6	1.96
Painful swallowing	46.	142(c)	27.8	1.99
Breast pain/changes	47.	140	27.5	1.59
Vomiting	48.	134	26.3	1.71
Prominent leg veins	49.	133	26.1	1.94
Sore/bleeding gums	50.	130(c)	25.5	1.73
Rectal itching	51.	130	25.5	1.97
Swollen glands	52.	127	24.9	1.92
Skinny arms and legs	53.	126(b)	24.7	1.99
Mouth ulcers	54.	115	22.5	1.80
Flushing	55.	115	22.5	1.73
Sores or lumps on genitals	56.	113	22.2	1.85
Concern over weight gain	57.	110	21.6	1.72
Nose bleeds	58.	107	21.0	1.84
Seizures/tremors	59.	99	19.4	2.15
Nipple discharge	60.	61	12.0	1.68
Rectal discharge	61.	52	10.2	1.80
Blood in spit/sputum	62.	50	9.8	1.76
Hump on back of neck/shoulders	63.	47	9.2	1.95
Rectal bleeding	64.	46	9.0	1.95

"**a**" 1 (mild), 2 (moderate), 3 (severe). "**b**" Significantly more frequent among PLHIV on ART (P > .01), "**c**" Significantly more frequent among PLHIV not on ART (P > .01)

Table 2 shows demographic, social and health status information and a multiple hierarchical regression analysis with HIV symptom intensity as the dependent variable. Most PLHIV (78.3%) were women, 75.6% were between 26 to 45 years old, and almost all were Black African (96.9%). Half of the PLHIV came from rural (48%) and another half from urban areas (52%) and at least 80 PLHIV came from each of the seven districts in Eastern Cape except for Ukhahlamba (n = 41). Three in four PLHIV (74%) had more than Grade 7 education, 70.5% were never married, and the average number of own children was 2.0 (SD = 1.7). On the question if they had gone without enough food to eat in the past 12 months 40.6% responded never or rarely, 47.3% sometimes and 12% often. Two in five of the PLHIV (39%) indicated that they had ever been on a disability grant for HIV/AIDS, 35% are currently on a disability grant for HIV/AIDS and 13% said that they were stopped on from receiving the disability grant for HIV/AIDS (since they no longer fulfilled the criteria). Three in five (60%) PLHIV were aware of their HIV diagnosis within the last four years and 66% classified themselves with being given an AIDS (advanced stage of HIV) diagnosis, in particular in Cacadu, Chris Hani and Alfred Nzo districts. One in three (32%) PLHIV self-reported a CD4 count of less than 200 and 48% were currently on ART. Two in three PLHIV (63.7%) had visited a clinic three or more times in the past three months and 17% had been at least once hospitalized in the past three months.

**Table 2:** Demographic, social and health status information and predicting HIV symptom intensity from demographic, social and disease characteristics

Independent variables	N	M (SD)	Regression equation		
		Symptom intensity	1 (adjusted R square = .08)	2 (adjusted R square = .16)	3(adjusted R square = .20)

*Gender*			Beta	Beta	Beta
Male	104	24.6 (12.3)	.07	.01	.02
Female	406	25.9 (13.9)			
*Age*					
< 35 years	352	24.9 (13.4)	.05	.13*	.07
35 years and more	253	27.7 (14.0)			
*Educational level*					
Grade 7 and less	158	30.0 (14.5)	-.20	-.18**	-.07
Grade 8–11	296	26.1 (13.3)			
Grade 12 and above	152	22.2 (12.6)			
*Geolocality*					
1. Rural	255	22.6 (13.4)	--	--	--
2. Informal settlements (shanty towns or squatter camps)	40	29.9 (13.1)	.13***	.08	.14
3. Urban	212	28.3 (13.3)	.20***	.19**	.05
*Do you have health insurance?*					
Yes	53	23.9 (13.6)		.11*	.17*
No	455	25.8 (13.3)			
*Often or sometimes not enough food in past 12 months*					
Yes	292	29.0 (13.5)		.14*	.28***
No	211	21.9 (12.8)			
*Disability grant for advanced HIV*					
Yes	213	25.5 (13.1)		-.19***	-.12
No	111	31.3 (15.0)			
*Disability grant stopped*					
Yes	78	30.5 (15.2)		.06	.15
No	209	25.4 (13.2)			
*Have you been given an AIDS diagnosis?*					
Yes	349	27.5 (13.0)			-.00
No	157	21.6 (13.9)			
*Hospitalized in past 3 months*					
Yes	81	29.7 (13.7)			.11
No	425	24.9 (13.5)			
*Most recent CD4*					
< 200	196	28.4 (13.4)			-.12
200–350	145	26.2 (13.8)			
> 350	169	23.7 (14.1)			
*ART*					
Yes	292	26.0 (13.0)			.27***
No	297	26.2 (14.3)			
*ART*					
One year and more	123	25.2 (13.4)			-.13
None or less than one year	109	29.2 (14.1)			
*Taking traditional medicine for HIV*					
Yes	40	26.2 (13.5)			-.11
No	549	26.3 (15.8)			

***P < .001, **P < .01, *P < .05

The first regression identified residence in urban and informal settlements but not sex, age and educational level as independent predictors for HIV symptom intensity. In the second regression analysis four social variables were added, and age, lower education, urban residence, having a health insurance, not having enough food and being on a disability grant for HIV were identified as predictors for HIV symptom intensity. In the third regression six disease characteristics variables were added, and only not having enough food, being on ART and having a health insurance were found as predictors for HIV symptom intensity (see Table 2).

## Discussion

This study found among a sample of PLHIV in the Eastern Cape province of South Africa that they were extremely ill. The sample reported a total of 64 different symptoms with a mean of 26 symptoms per person which is much higher than in a study of 743 men and women living with HIV/AIDS from different Southern African countries in 2002 (17.6 symptoms per person) [10]. Participants also reported high levels of psychological problems such as fear or worries (rank 9), depression (sadness) (rank 13), and anxiety (rank 23), which was similar to what was found in the Southern African study (fear or worries (rank 4), depression (sadness) (rank 11), and anxiety (rank 29) [10]. Hughes [14] also found that common under recognized and/or under treated symptoms that may influence the quality of life of PLHIV include fatigue, pain, anxiety/depression, and sleep disturbances. Fatigue was found to be highly prevalent symptom, as found in a study among men and women living with HIV/AIDS in Southern Africa [15]. People living with HIV seek to acknowledge that fatigue is a legitimate concern, not only by health care professionals, but also people with whom they live. It is imperative that health care providers who work with people living with HIV-related fatigue consider the wider social aspects of the person's life as well as physical symptoms [16].

Pain in the form of headache (79%), painful joints (65%), muscle aches (59%), abdominal pain (55%), chest pain (46%), and breast pain (28%) was one of the most frequently experienced symptoms among PLHIV in this study. Similarly, Norval [16] investigated symptoms of AIDS patients in Soweto and found that of the 103 respondents (of which 3.9% had access to ART), 98% experienced pain and 34% mentioned pain as the worst overall symptom; lower limb pain was the most prevalent pain (66%), followed by mouth pain (51%), headache (42%), throat pain (40%) and chest pain (18%). People living with HIV/AIDS often have multiple pains occurring concurrently and are often neglected or poorly managed symptom of AIDS [17]. The findings document that, despite improved treatments for HIV/AIDS, many living with the disease continue to experience high levels of physical and psychological symptomatology. From a clinical perspective, managing treatment for a patient with 26 symptoms is highly complex, often involving detailed clinical assessment and review, polypharmacy, assessment of drug and symptom interactions, and the potential for compound side effects [18]. High levels of symptomatology may also impact patients' ability or willingness to adhere to various treatment regimens. In line with the finding that higher symptoms were associated with having been given an AIDS diagnosis, Singer et al. [19] found that painful illnesses were reported at all stages of systemic disease but were more common in the later stages of disease and in subjects who progressed to a more advanced stage during the study period. The prevalence of many of these symptoms was similar to (and in some instances worse than) that of terminally ill cancer patients [20,21].

The study results suggest that social factors such as lower educational levels, lack of enough food and not on a disability grant are significant determinants in the presentation of HIV-related symptoms. Similarly, Mathews et al. [8] in a nationally representative US-based sample of HIV-infected adults receiving medical care found that HIV symptoms were greatest, among others, in persons with lower educational levels and lower income. Poor nutritional status is known to be reflected in signs and symptoms related to HIV infection. The effects of malnutrition on the immune system are well known and include decreases in CD4 Tcells, suppression of delayed hypersensitivity, and abnormal B-cell responses [22]. Almost half of the PLHIV (47.3%) in this study reported sometimes and 12% reported often to have gone without enough food to eat in the past 12 month, which was associated with higher symptom frequency. With limited access to adequate nutrition and intake of sufficient calories become even more critical to stabilize symptoms and prevent disease progression and early death. Nutritional interventions can help manage symptoms, promote response to medical treatment, slow progression of the disease, and increase the quality of life by improving daily functioning. Nutritional interventions should be considered alongside longer term, multisectoral interventions that work to improve food security and living conditions. The study reveals that HIV symptom levels were associated with not being on a disability grant. This may be attributed to the findings that Phaswana-Mafuya and Peltzer [23] reported from this sample elsewhere that PLHIV used the grant to meet basic household and health care needs. This is not surprising given the fact that more than half (53.4%) of the respondents were unemployed and looking for work, 15% felt sick or disabled and unable to work, and only 8.4% were (full-time or part-time) employed. The disability grant was a source of health and welfare for respondents in the sample and this may be true for most HIV sufferers in the Eastern Cape as the province has the highest unemployment rate in the country (48.5%) and 63% poverty rate – the second highest in the country [24]. The expansion of the social safety net and protecting poor people from vulnerability as well as accessing the different social assistance grants by the poor is a challenge for the country at large [23].

Regarding various disease characteristics (self-reported AIDS diagnosis, hospitalization, CD4 cell counts, ART and traditional medicine); only being on ART was in this study associated with HIV symptom intensity. Surprising was that being on ART was not associated with lower HIV symptom levels. Similarly, Brechtl et al. [25] found that among the various psychological and symptom distress measures, only depression ratings (based on the Hamilton Depression Rating Scale) decreased significantly after three months of HAART. There were no significant changes in any other symptom distress measures. Likewise, there was no significant decrease in the number of symptoms endorsed on the MSAS, ratings of patients' overall physical functioning (KPRS scores), or quality of life as measured by the EFAT. Among patients who reported pain, there was no significant change in the level of pain intensity or pain-related functioning interference. HAART regimens appear to have positive effects on CD4 count, HIV viral load, and several other measures of physical well-being in patients with advanced AIDS. However, the benefits of HAART on pain and symptom distress, and psychological well-being over a short period of time are less clear. This finding certainly raises concerns regarding the projected applicability of HAART regimens among some patients with advanced HIV disease. Further research is clearly necessary to better understand the benefits of HAART therapy in patients with advanced HIV infection. Vogl et al. [17] found that the number of symptoms was highly associated with poorer quality of life. Contrary to the current study, they found that CD4+ T-cell count was not associated with symptom number.

### Limitations

Because non-probability sampling was used, the findings are not generalisable to the entire population of PLHIV in the Eastern Cape Province. Another limitation of the study is that CD4 cell counts were assessed by self-report data and not confirmed with clinical records. Even though 32.3% of patients reported a CD4 cell count less than 200, the group as a whole seem to be "extremely ill"; 66.4% self-reported to have been diagnosed with AIDS. This may reflect the limitation in self reporting of CD4 counts and/or AIDS diagnosis because the data fail to reflect how ill the patients are [11].

## Conclusion

Because of the high levels of symptoms reported, the results imply an urgent need for effective health care, home- and community-based as well as self-care symptom management to help patients and their families manage and control AIDS symptoms [10,26,27]. Further research is needed on symptoms related to HIV infection itself, those related to opportunistic infections, and those related to medications and treatments, in particular during the course of the illness.

## Abbreviations

ARV: Antiretrovirals; ART: Antiretroviral Treatment; CD4: Cluster of differentiation 4; PLHIV: People Living with HIV.

## Competing interests

The authors declare that they have no competing interests.

## Authors' contributions

KP and NPM conceptualized and designed the study, analysed and interpreted the data, drafted and revised the manuscript. All authors read and approved the final draft of the manuscript.

## Pre-publication history

The pre-publication history for this paper can be accessed here:


